# Unveiling of Concealed Processes for the Degradation of Pharmaceutical Compounds by *Neopestalotiopsis* sp.

**DOI:** 10.3390/microorganisms7080264

**Published:** 2019-08-16

**Authors:** Bo Ram Kang, Min Sung Kim, Tae Kwon Lee

**Affiliations:** Department of Environmental Engineering, Yonsei University, Wonju 26493, Korea

**Keywords:** Ascomycota, biodegradation, extracellular enzymes, *Neopestalotiopsis*, pharmaceutical compounds

## Abstract

The presence of pharmaceutical products has raised emerging biorisks in aquatic environments. Fungi have been considered in sustainable approaches for the degradation of pharmaceutical compounds from aquatic environments. Soft rot fungi of the Ascomycota phylum are the most widely distributed among fungi, but their ability to biodegrade pharmaceuticals has not been studied as much as that of white rot fungi of the Basidiomycota phylum. Herein, we evaluated the capacity of the soft rot fungus *Neopestalotiopsis* sp. B2B to degrade pharmaceuticals under treatment of woody and nonwoody lignocellulosic biomasses. Nonwoody rice straw induced laccase activity fivefold compared with that in YSM medium containing polysaccharide. But B2B preferentially degraded polysaccharide over lignin regions in woody sources, leading to high concentrations of sugar. Hence, intermediate products from saccharification may inhibit laccase activity and thereby halt the biodegradation of pharmaceutical compounds. These results provide fundamental insights into the unique characteristics of pharmaceutical degradation by soft rot fungus *Neopestalotiopsis* sp. in the presence of preferred substrates during delignification.

## 1. Introduction

The conventional wastewater treatment process is typically designed to remove organic carbon, nitrogen, and phosphorous, and only a few wastewater treatment plants are equipped with advanced tertiary treatment process [1]. In this sense, many pharmaceutical products presenting in wastewater are not fully removed and are then released with the effluent into the aquatic environment [2,3]. The presence of pharmaceutical products has raised emerging concerns due to their cumbersome biorisks even at low concentration (ng L^−1^ to μg L^−1^).

Fungi are among the most diverse groups of microorganisms. Given their widespread decomposing potential, they may offer promising degrading abilities for organic compounds, ranging from lignocellulose biomasses to recalcitrant pharmaceutical products [4]. These compounds were efficiently degraded by fungal species through several mechanisms, including bioprecipitation, bioadsorption, and biodegradation [5,6,7]. Some fungal species have developed repertoires of lignocellulolytic enzymes, including peroxidases, laccases, and oxygenases, which allow degradation of highly recalcitrant molecules [8]. Lignocellulolytic enzyme-producing fungi are categorized as white rot fungi, brown rot fungi, or soft rot fungi. White rot fungi, which include 90% of wood-rotting fungi, mainly attack lignin components in wood, whereas brown and soft rot fungi metabolize cellulose and hemicellulose [9,10]. White rot fungi are generally composed with a phylum of Basidiomycota, which have primarily drawn interest in biodegradation studies because they can enzymatically degrade a variety of substrates [11].

In contrast with Basidiomycota, most of Ascomycota comprise soft rot fungi and are the largest division in fungi, comprising >60% of all fungi on earth [12]. Despite their wide distribution, the ability of Ascomycota species to degrade xenobiotics and pharmaceutical compounds remains poorly understood [13]. Hitherto, no model Ascomycota organisms have been considered in studies of biodegradation of organic pollutants, in contrast with the Basidiomycota species *Trametes versicolor* and *Phanerochaete chrysosporium* [5]. Ascomycota are decomposers with versatile repertoires of extracellular and intracellular enzymes and great potential for lignin depolymerization [14]. Lignocellulolytic enzyme production causes lignin decomposition and has been recorded in certain organisms of the genera of Xylariales order [15]. The Ascomycota species *Trichoderma reesei* secretes distinguishingly large amounts of lignocellulolytic enzymes [16]. Ma et al. [17] also demonstrated that Ascomycota fungi play key roles in the decomposition of straw residues in arable soils and have been associated with balanced soil health. Studies of lignin-degrading enzymes from Ascomycota species are recent, and the application of these enzymes to the biodegradation of pharmaceutical compounds has scarcely been considered.

The goal of this study was to determine the lignocellulolytic potential of Ascomycota species and assess their abilities to degrade pharmaceutical compounds using their lignocellulolytic enzymes. We screened and identified wood-rotting fungi producing the lignocellulolytic enzymes efficiently from forest environments. We evaluated biochemical characteristics of lignocellulosic enzymes in the presence of lignocellulosic biomasses, and assessed the degradation of pharmaceutical compounds including acetaminophen, sulfamethoxazole, and carbamazepine. This study demonstrated the pharmaceutical biodegradation capacity of Ascomycota soft rot fungus, and indicated their limitations for applications to current environmental problems.

## 2. Materials and Methods 

All chemicals were of analytical grade. Acetaminophen, sulfamethoxazole, and carbamazepine were purchased from Sigma-Aldrich (St. Louis, MO, USA). Dextrose was purchased from Junsei (Tokyo, Japan). Potato dextrose agar (PDA), malt extract, peptone, and yeast extract were purchased from BD Difco™ (Heidelgerg, Germany). Methanol and acetonitrile were purchased from J.T.Baker (Phillipsburg, NJ, USA), and 2, 2′-azino-bis (3-ethylbenzthiazoline-6-sulfonic acid) (ABTS) and other chemicals were purchased from Sigma-Aldrich.

### 2.1. Sample Collection and Screening on Indicator Agar

Forty rotten wood branches were collected from Mae Mountain (37°17′33.8″ N 127°55′21.6″ E) and Dukga Mountain (37°17′02.2″ N 127°53′47.8″ E) in Wonju, South Korea. Small wood pieces (5 to 10 mm) from rotten wood branches were put on the middle of PDA and incubated until mycelium came out. Morphologically distinct mycelium were taken and transferred to new PDA plates until pure cultures were obtained. Pure isolates were then transferred to indicator agar containing ABTS according to the method of Crowe and Olsson [18]. Green-colored halo zones around the plugs appeared when the fungal species produced oxidative enzymes. Isolate with the longest halo-zone diameters from triplicate experiments was selected for subsequent experiments.

### 2.2. DNA Extraction, PCR Amplification, and Phylogenetic Analysis

Fungal mycelia (10 to 20 mg) were disrupted using a 1.5 mL tube pestle. DNA was extracted using FastDNA™ SPIN KIT (MP Biomedicals, Santa Ana, California, USA) according to the manufacturer’s instructions. The primers ITS-1F (5′-CTTGGTCATTTAGAGGAAGTAA-3′) and ITS4 (5′-TCCTCCGCTTATTGATATGC-3′) were used to amplify the ITS region of rDNA [19,20]. PCR mixtures (25 μL) contained 16 μL of sterile nuclease-free water, 2.5 μL of 10× Ex Taq Buffer, 1 μL of genomic DNA, 2 μL of 2 mM dNTP, 1 μL of 25 mM MgCl_2_, 0.5 μL of each primer, 1 μL of 20 mg/mL bovine serum albumin, and 0.5 μL of Ex Taq (5 U/μL) (all reagents from Takara, Shiga, Japan). PCR analysis was performed according to the following program: 95 °C for 4 min, 36 cycles of 95 °C for 40 s, 54.5 °C for 40 s, 72 °C for 1 min, with a final elongation step at 72 °C for 4 min. PCR products from replicates were purified using QIAquick PCR Purification Kits (QIAGEN, Hilden, Germany). Sequence analyses of purified amplicons were performed by Macrogen (Seoul, Korea). Sequence was assigned using nucleotide BLAST (https://blast.ncbi.nlm.nih.gov/Blast.cgi) and similarities with reported sequences of other fungal species were assessed. Phylogeny was constructed based on the ITS data under the conditions of the Maximum Likelihood method as implemented in MEGA7 using Kimura 2-parameter model with 1,000 bootstrap replications [21]. The sequence has been deposited into GenBank with the accession number MK646009.

### 2.3. Tests of Optimal Conditions with Four Culture Media

Five agar plugs from fungal isolates incubated in PDA media for two days were inoculated into 250 mL Erlenmeyer flasks containing 100 mL of malt extract medium (ME) containing 20 g L^−1^ dextrose, 20 g L^−1^ malt extract, and 1 g L^−1^ peptone (pH 4.5). Fungal suspensions were obtained by homogenizing seven-day-old cultures and were stored in 0.85% saline solution at 4 °C until used as inoculums. Four fungal culture media possessing a variance of carbon sources were compared to determine optimum conditions for laccase production. These media were ME, Sabouraud dextrose (SD) containing 20 g L^−1^ dextrose and 10 g L^−1^ peptone, yeast malt extract medium (YM) containing 20 g L^−1^ dextrose, 3 g L^−1^ malt extract, 3 g L^−1^ yeast extract, and 5 g L^−1^ peptone, and YpSs medium (YSM) containing 15 g L^−1^ starch, 4 g L^−1^ yeast extract, 1 g L^−1^ KH_2_PO_4_, and 0.5 g L^−1^ MgSO_4_. Suspensions (1 mL) were inoculated into 250 mL Erlenmeyer flasks containing 100 mL of each medium and were incubated at 25 °C for seven days on a shaker at 120 rpm. Culture suspensions were transferred into microtubes and supernatants were collected by centrifugation at 6,000 *g* at 25 °C for 5 min. Supernatants were used directly for determinations of laccase activities.

### 2.4. Incubation with Lignocellulosic Biomasses

Woody (ash, fir, and oak) and nonwoody (rice bran, rice straw, and soy flour) sources were purchased from the domestic online market. Ash, fir, oak, and rice straw samples were cut into fine sections (0.7–1.0 cm). All sources of lignocellulosic biomass were washed with distilled water, were dried at 60 °C for 24 h, and were then used as substrates in place of starch in YSM medium (woody sources, 20 g L^−1^ and nonwoody sources, 10 g L^−1^). Enzyme activity was determined after inoculating 1 mL fungal suspensions into 250 mL Erlenmeyer flasks containing 100 mL aliquots of each media. Fungal isolates were then incubated at 25 °C for seven days at 120 rpm. Supernatants were directly used for determinations of laccase activities.

### 2.5. Determination of Lignin and Sugar Concentrations

Lignin portions of each biomass were quantified using the acetyl bromide method as described previously [22]. Determinations of sugar contents were performed using dinitrosalicylic (DNS) acid reagent [23] and the method was modified for 96-well plates. Briefly, 300 μL samples were reacted with single volumes of DNS reagent containing dinitrosalicyclic acid at 10 g L^−1^, sodium sulfite at 0.5 g L^−1^, and sodium hydroxide at 10 g L^−1^ at 90 °C for 10 min, and 100 μL aliquots of 40% potassium sodium tartrate solution were then added. Absorbance levels were then recorded in supernatants at 575 nm.

### 2.6. Enzyme Activity Measurements

Laccase activities were measured by spectrophotometrically monitoring the oxidation of 0.5 mM ABTS to its radical cation at 420 nm in 0.1 M acetate buffer at pH 4.5 (ε_420_ = 36,000 M^−1^ cm^−1^) [24]. Lignin peroxidase (LiP) activities were calculated from the rate of oxidation of 2 mM veratryl alcohol at 310 nm in 0.1 M tartrate buffer (pH 3) containing 0.4 mM H_2_O_2_ (ε_310_ = 9,300 M^−1^ cm^−1^) [25]. Manganese peroxidase (MnP) activities were determined according to the oxidation of 7 mM MnSO_4_ at 238 nm in 0.1 M tartrate buffer (pH 5) containing 0.05 mM H_2_O_2_ (ε_310_ = 6,500 M^−1^ cm^−1^) [26]. One unit of activity (U) was defined as the amount of enzyme required to oxidize 1 μmol of product per min.

### 2.7. Micropollutant Analysis

Five-milliliter aliquots of supernatant containing laccase were applied to 0.2 μm filters under sterile conditions. Stock solutions (1 g L^−1^) of acetaminophen (ACN), carbamazepine (CMZ), and sulfamethoxazole (SMX) were diluted to final concentrations of 20 mg L^−1^ in Mcllvaine buffer (pH 4.0). The final concentration of methyl alcohol in reaction mixtures was 9% (*v*/*v*) and was sufficient to prevent precipitation of compounds. To 2 mL aliquots of Mcllvaine buffer containing three compounds in 10 mL glass vials, 200 μL aliquots of filtered supernatants were added as enzyme sources. Heat-inactivated enzyme was used as a negative control. Vials were incubated in the dark at 25 °C for 24 h and enzyme activity was then stopped by injection of the enzyme inhibitor sodium azide at 10 μM.

Pharmaceutical products were detected using a reverse-phase liquid chromatography instrument equipped with a photo diode array detector (HPLC-PDA) and a YMC-Triart C18 column (4.8 mm × 250 mm; particle size, 3.5 μm; YMC, Kyoto, Japan). Eluents (A) ultrapure water and (B) acetonitrile were applied at a flow rate of 0.5 mL min^−1^. The following gradient profile was applied as described by Gineys et al. [27]: linear increase from 10% B to 80% B over 25.45 min, isocratic elution for 2 min, and restoration of initial conditions within 1 min. The volume of injection was 50 μL and products were detected spectrophotometrically at 230 nm.

### 2.8. Statistical Analysis

All statistical analyses were performed in R (version 3.5.1) [28]. Duncan’s multiple range tests were used to identify significant differences between groups at *p* < 0.05 through R package ‘agricolae’ (version 1.3.1). Substrate specificity of the target strain was assessed by calculating the Spearman correlation coefficient for sugar and laccase concentrations.

## 3. Results and Discussion

### 3.1. Screening and Identification of Isolated B2B

To screen fungal species that carry lignocellulolytic systems, we generated 25 fungal isolates from rotten wood branches. Nine isolates grew on indicator agar and formed green-colored halo zones around their plugs. The diameters of halo zones ranged from 14 to 35 mm after three days (Table S1). Isolate B2B had the widest halo zone (35 mm), indicating the highest rate of laccase production. This strain was consequently selected for further experiments.

**Figure 1:**
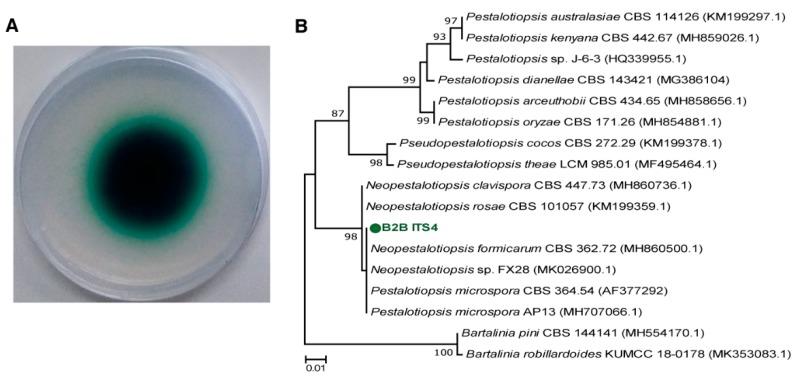
Laccase activity of B2B on ABTS agar plates using colorimetric assays (**A**) and a phylogenetic tree for B2B and related strains based on ITS gene sequences (**B**). Green-colored halo zone on the agar indicates the contributions of laccase. The scale bar corresponds with 0.01 substitutions per nucleotide position.

Based on ITS rRNA gene analyses, B2B were close to *Neopestlosiopsis* sp. belonging to Xylariales order, which is one of the largest of the Ascomycota phylum [29] (Figure 1B). Recently, *Neopestalotiopsis* sp. demonstrated the capacity of dye decolorization using its oxidative enzymes [30]. The enzyme system or applications of *Neopestalotiopsis* are poorly characterized compared with other closed genera *Pestalotiopsis*, which is regarded as having a promising application for industrial fields using valuable metabolites [31] Chen et al. [32] isolated *Peslatoptiopsis* strain J63 and showed robust laccase activity in the presence of rice straw during solid-state fermentation. Russel et al. [33] previously identified two *Pestaloptiopsis microspora* strains that have greater degradation capacity for synthetic polyurethane than *Aspergillus niger*. These results may warrant consideration of *Neopestlosiopsis* sp. as a degrader of recalcitrant organic pollutants.

### 3.2. Effects of Cultivation Conditions on Laccase Production

**Figure 2:**
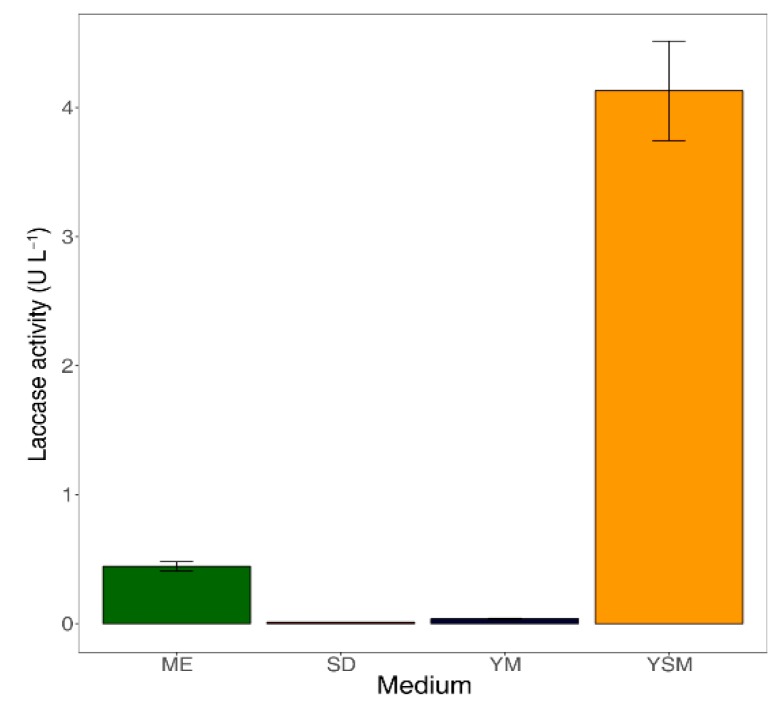
Laccase activities in culture supernatants after seven days of incubation. The error bars represent the standard deviation from triplicates. ME: Malt extract medium, SD: Sabouraud dextrose, YM: Yeast malt extract medium, YSM: YpSs medium.

Enzyme production by fungal species is significantly influenced by culture conditions [34]. In particular, lignocellulolytic enzyme production by wood-rotting fungi was sensitive to substrates in the media [35]. To assess the substrate specificity of B2B, we determined laccase enzyme production in four media that contained differing carbon and nitrogen sources. Enzyme production increased significantly when B2B was incubated in YSM (4 U L^−1^) and ME (0.5 U L^−1^) after seven days, yet negligible enzyme production was observed in SD and YM media (Figure 2). Moreover, lignin peroxidase and manganese peroxidase were not detectable in any of the present culture media.

Laccase was only produced efficiently in YSM medium, and this may reflect substrate specificity. Fungal laccases are generally produced from secondary metabolic processes during pigmentation, morphogenesis, and delignification [36]. YSM may facilitate laccase production due to its starch contents, which were the most abundant of polysaccharides in this medium. Although polysaccharides have not been shown to have direct effects on laccase production, they reportedly play important roles as low-molecular-weight mediators during delignification [37]. Previous studies also show that disaccharides improve laccase production [38]. Our results similarly show that laccase is produced in ME medium, which contains disaccharides. In contrast, laccase has been shown to be inhibited when monosaccharides, such as glucose, are present at sufficient concentrations [39]. Xiao [40] associated decreasing laccase activities in glucose-based cultures with repression of genes that encode metabolic enzymes for various carbon sources. In agreement, our results confirmed that laccase is not produced in SD and YM media containing the monosaccharide glucose. These results indicate that the complexity of sugar structures has a major impact on laccase production by B2B.

### 3.3. Effects of Six Lignocellulose Sources on Laccase Production

Lignin-derived aromatic compounds are generally added to fungal cultures to enhance laccase production as inducers [41]. These inducers increased yields of the enzyme and improved enzyme stability [42]. Yet, most of these inducers are chemically well refined and their high costs prohibit application to nonlaboratory conditions. To overcome this limitation, we determined laccase production in the presence of natural inducers such as lignocellulosic biomasses. We tested six lignocellulosic biomasses, including those from three woody (ash, fir, and oak) and nonwoody (rice bran, rice straw, and soy flour) sources, as substrates and laccase inducers.

In these experiments, the highest laccase activity was observed when B2B was incubated with rice straw (21.4 U L^−1^). This value was five times higher than that observed with YSM, which contains starch (Table 1). Rice bran and soy flour also induced laccase activities to 16.2 and 9.6 U L^−1^, respectively. These results confirmed that B2B grows with lignocellulosic biomasses as sole substrates, and that these sources enhance laccase production. All of the present biomasses that increased laccase activities, however, were from nonwoody sources. Yet, some woody sources showed modest effects on laccase production and B2B scarcely produced laccase when incubated with ash or oak (0.3 and 0.1 U L^−1^, respectively). Sugar contents in culture supernatants were also much greater in cultures with woody sources (around 85 mg L^−1^ from fir and oak, 122.2 mg L^−1^ from ash) than in those with nonwoody sources (65 to 70 mg L^−1^).

Greater laccase production in the presence of nonwoody biomasses may reflect differences in the composition of lignin units and corresponding substrate preferences of B2B. Lignin structures comprise the three phenylpropanoid units syringyl (S), guaiacyl (G), and p-hydroxyphenyl (H) [43]. Moreover, whereas lignins from hard wood (ash and oak in this study) comprise G and S units, those from soft wood (fir in this study) predominantly comprise G units. In contrast, lignins from nonwoody sources generally contain H units, which are barely detectable in woody lignocellulosic biomasses. B2B likely prefers to produce laccase in response to the decomposition of H-lignin portions of nonwoody sources. These H-lignins have simpler structures than the G or S lignin units from woody sources. Despite the high proportions of lignin in woody sources, our observations of high laccase production in the presence of nonwoody sources support this phenomenon (Table 1). In addition, laccase activities were positively associated with lignin portions (*r* = 0.9746, *p* < 0.05) in nonwoody sources, but these variables were not correlated in woody sources (*r* = 0.1285, *p* > 0.05). As mentioned in Section 3.2 above, B2B was sensitive to substrates for laccase production in the presence of sugar. Although we did not classify the sugar types in this study, we did observe higher sugar concentrations following lignocellulolysis in the presence of woody sources (Table 1). Sugar concentrations were also negatively correlated with laccase production in both woody (*r* = −0.8796, *p* < 0.05) and nonwoody sources (*r* = −0.3128, *p* > 0.05), and these correlations were highly significant in the presence of woody sources. Martinez [44] compared wood degradation patterns of white rot and soft rot using Py-GC/MS, which distinguishes between polysaccharide and lignin regions. These studies confirmed that B2B prefer to decompose polysaccharides over lignin. Hence, when lignin and polysaccharides are both present as substrates, B2B may selectively metabolize polysaccharides to produce reducing sugars. These results suggest that laccase production from B2B is regulated by types of lignin units in lignocellulosic biomasses or by intermediates of delignification.

### 3.4. Removal Characteristics of Target Pharmaceuticals

**Figure 3:**
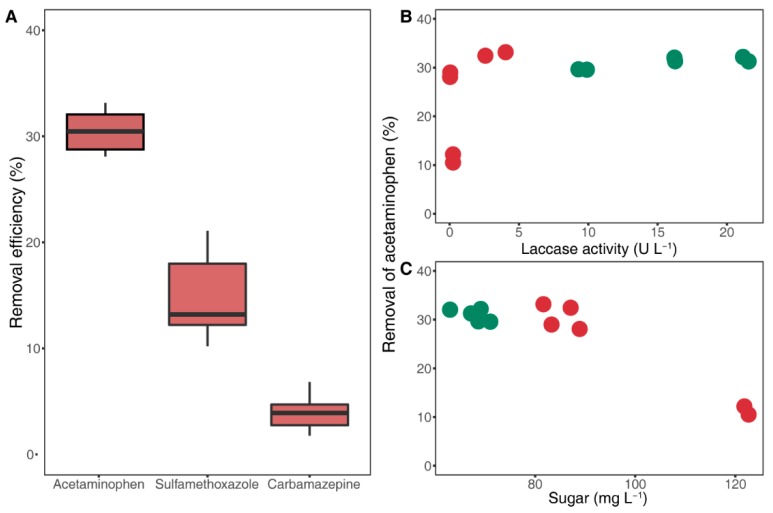
Pharmaceutical removal efficiency of laccase (**A**) and removal of acetaminophen related with laccase activities (**B**), and sugar concentrations (**C**); decreases in acetaminophen, sulfamethoxazole, and carbamazepine concentrations due to the activities of crude laccase from seven-day-old cultural supernatants. The green and red colors indicate nonwoody and woody lignocellulosic biomasses in (**B**) and (**C**), respectively.

To evaluate the potential of B2B to degrade pharmaceutical products, we selected three products with different electron-donating groups. Regardless of lignin substrates, 30%, 15%, and 3% of ACN, SMX, and CMZ, respectively, were degraded on average, whereas ACN removal was <15% in the presence of lignin from ash (Figure 3). These results suggest high substrate specificity of laccase toward substituents such as phenol (–OH) and amine (–NH_2_) groups [34], because unlike SMX (only amine) and CMZ (none of both groups), ACN carries both of these functional groups.

Unexpectedly, the degradation process did not follow a general enzymatic reaction, as indicated by disproportionate degradation efficiency and laccase production. Specifically, 28% to 33% of ACN was removed from five lignocellulosic biomasses, irrespective of variances in laccase activities (Figure 3B). Yet, with ash, for which laccase activity was slightly higher than that for oak, the degradation rate was exceptionally low at about 10%. These results indicate that laccase is not solely responsible for ACN degradation, but is also regulated by external factors.

Laccase contains the three type domains of copper center T1 Cu (single-electron oxidation of phenolic substrates) and T2/T3 Cu (four-electron reduction of O_2_) [45]. Following oxidation of substrates at the T1 Cu site, electrons are transferred to the T2/T3 Cu site and O_2_ is reduced to H_2_O [9]. A recent study demonstrated that byproducts of delignification by laccase-like enzymes, such as H_2_O_2_, inhibit laccase activities by interfering with active sites [46]. Some fungal species produce H_2_O_2_ during saccharification in the presence of polysaccharides, and H_2_O_2_ could inhibit laccase activity by inactivating the T2 Cu site [47,48]. We suggest that the formation of sugars by B2B was an indirect consequence of delignification and saccharification. Although we did not directly measure H_2_O_2_ concentrations, laccase activities may be regulated by H_2_O_2_ that is produced from saccharification.

Our results accordingly show no significant differences in degradation rates of pharmaceuticals at similar sugar concentrations (from 65.9 to 86.1 mg L^−1^), yet degradation rates were drastically reduced at high concentrations (over 120 mg L^−1^) in the presence of ash treatments (Figure 3C). These results suggest that sugars, as intermediate products, indirectly indicate the effects of saccharification on the degradation efficiency of laccase toward pharmaceutical products.

## 4. Conclusions

Herein, the characteristics of laccase production by the soft rot fungus *Neopestalotiopsis* sp. B2B were examined in the presence of culture media containing differing carbon types and lignocellulosic biomasses. We also evaluated the biodegradability of three selected pharmaceutical products by laccase induced by biomasses. Under these conditions, enzyme production varied significantly depending on the complexity of the sugar structures and the compositions of lignin units in the biomasses. The present pharmaceutical compounds were not degraded in proportion with laccase activities, with only approximately 30% reductions in concentrations. These results suggest that intermediate products of laccase-mediated saccharification inhibit laccase activity by interfering with its active sites. This study provides fundamental information about pharmaceutical degradation by soft rot fungal cultures and its lignocellulolytic enzymes. Our results may inform future developments of fungal-based biological treatment systems for pharmaceutical removal. 

## Figures and Tables

**Table 1:** Lignin components of six different lignocellulosic biomasses, and corresponding laccase activities and sugar concentrations in cell culture supernatants. The effects of two types of lignocellulosic biomasses on laccase activities were tested. Lignin components were correlated with laccase activities and sugar concentrations from woody and nonwoody lignocellulosic biomasses, separately. Data are presented as means ± standard deviations from triplicates.

Types	Sources	Lignin Components (mg Lignin/g Cell Wall)	Laccase Activity (U L^−1^)	Lignin vs Laccase		Concentration of Sugar (mg L^−1^)	Lignin vs Sugar	
				Correlation Coefficient	*p*-Value		Correlation Coefficient	*p*-Value
Woody	Ash	11.8 (± 2.9)	0.3 (± 0.0)	0.1285	0.8083	122.2 (± 0.4)	−0.8796	0.0208
	Fir	12.6 (± 2.0)	3.3 (± 1.0)			84.4 (± 2.7)		
	Oak	15.2 (± 0.5)	0.1 (± 0.0)			86.1 (± 4.0)		
Nonwoody	Rice bran	6.8 (± 3.5)	16.2 (± 0.0)	0.9746	0.0009	65.9 (± 3.0)	−0.3128	0.546
	Rice straw	8.8 (± 1.6)	21.4 (± 0.3)			68.2 (± 1.0)		
	Soy	0.3 (± 0.1)	9.6 (± 0.4)			69.9 (± 1.2)		

## Supplementary Materials

The following are available online at https://www.mdpi.com/2076-2607/7/8/264/s1.
