# Supplementary material for: Unveiling of Concealed Processes for the Degradation of Pharmaceutical Compounds by Neopestalotiopsis sp
Source: Microorganisms. 2019 Aug 16;7(8):264. doi: 10.3390/microorganisms7080264 (PMC6722755; doi:10.3390/microorganisms7080264)
Supplement: Supplementary file 1 [file microorganisms-07-00264-s001.pdf]

microorganisms

MDPI

**Table S1.** Sampling site, strain, and diameters of green-colored halo zone from each strain on ABTS agar plates. Diameters of halo zone indicate the laccase activity. Data are presented as means ± standard deviations from triplicates.

| Sampling Site | Strain | Diameter (mm) |
|---|---|---|
| Mae Mountain | B1A | 33 (±1) |
| | B2B * | 35 (±2) |
| | B4A | 31 (±1) |
| | B7A | 32 (±2) |
| Dukga Mountain | D1A | 14 (±0) |
| | D3A | 23 (±1) |
| | D4A | 31 (±2) |
| | D17C | 24 (±1) |
| | D17B | 26 (±2) |

* B2B was finally selected for further study.
